# Clinical Reports

**Published:** 1864-01

**Authors:** N. S. Davis

**Affiliations:** Prof. of Practical and Clinical Medicine


ARTICLE V.

### CLINICAL REPORTS.

### CEREBRO-SPINAL MENINGITIS—LETTER FROM A CORRESPONDENT—CLINICAL REMARKS IN REPLY; BEING THE SUBSTANCE OF A LECTURE TO THE CLASS IN THE CHICAGO MEDICAL COLLEGE.

By N. S. DAVIS, M.D., Prof. of Practical and Clinical Medicine.

By cerebro-spinal meningitis, we mean an inflammation of the membranes and surface of the base of the brain, medulla oblongata, and upper part of the spinal cord. Ordinary inflammation of this portion of the nervous system is not of frequent occurrence in general practice, although occasionally met with, both in adults and children. When it does occur it is always dangerous, and often speedily fatal to the patient. This arises from the direct connection of this portion of the nervous centres, with the most important functions of animal life, such as respiration, circulation, and deglutition. An attack is generally ushered in by chilliness, followed by general febrile reaction; pain in the occipital region, often extending to the back of the neck and shoulders; stiffness or rigidity of the muscles of the neck and jaws; sometimes cramps in the muscles of the arms, with difficulty of deglutition; a contracted, frequent, and variable pulse; hurried respiration; and, as the disease advances, delirium ending in coma. While sporadic cases of this inflammation are not of frequent occurrence, a modified form of it has often occurred as an epidemic, in circumscribed localities. When thus occurring epidemically, it has been found to have been either closely associated with the prevalence of erysipelas, or connected with the same circumstances that usually originate typhus and pyæmia, namely, close and ill-ventilated rooms, over-crowded and uncleanly camps, &c.

We are occasionally informed of its occurrence and alarming fatality in very limited country districts.

Close inquiry, in most of such cases, will reveal the fact that those attacked have been sleeping in very small, or altogether over-crowded rooms, without any ventilation whatever. Such was found to be the case with some families in a neighborhood near Valparaiso, Indiana, in which the disease appeared and proved rapidly fatal, soon after the intense cold weather that ushered in the present month.

Only two days since, I received the following letter from a medical friend at Manckport, Harrison County, Ind.:—

"PROF. N. S. DAVIS,

"*Dear Sir:*—Knowing that you have the opportunity to inform yourself concerning all forms of disease, I drop you a letter to ask your views in regard to a disease that is prevailing in this county, to an alarming extent. So far as my knowledge extends, every case that has occurred, up to the present time, has proved fatal. The person attacked, complains of a slight cold for about 24 hours, when a moderate chill occurs, lasting from one to two hours.

"This is immediately followed by pain in the back of the neck, spine, and limbs; stiffness or rigidity of the muscles of the neck and jaws; and soreness or morbid sensibility of the surface, even to the ends of the fingers and toes.

"In half an hour the jaws become closed, with loss of speech, followed in a short time more, by complete unconsciousness. Death usually follows the coma, in from two to three hours. Please give me some information concerning this disease, if your time will permit, and greatly oblige, yours truly,

"H. K. DEEN."

The description here given, though brief, is sufficient to identify the disease as a cerebro-spinal meningitis.

The pain extending from the occipital region down the spine, with rigidity of the muscles of the neck and jaws, morbid sensibility or soreness of the flesh, especially of the extremities; followed so speedily by unconsciousness and death, point unmistakably to the cerebro-spinal axis as the seat of the disease. The rapidity with which the disease progresses to its final termination, is one of its most striking features. Thus the distinct chill or chilliness that marks the onset of severe symptoms is often followed by death in from six to twelve hours. But the *post mortem* examinations made by Dr. UPHAM and others, reveal not only the appearances of inflammation in the membranes enveloping the medulla and base of the brain, but more or less purulent or sero-purulent effusion. The rapid progress of the disease; the exceedingly brief period in which the suppurative process is established; with the sudden and generally fatal exhaustion of the patient; all indicate that the inflammation is of a strongly asthenic or septic character. This view of its nature is further indicated by the fact that the disease has often been associated with epidemic erysipelas; with the foul air of crowded military camps; and with small, unventilated lodging rooms in country districts.

If this view of the special character of the inflammation in epidemic cerebro-spinal meningitis is correct, it enables us readily to understand why the treatment by antiphlogestic and sedative measures on the one hand, or by simple stimulants and tonics, on the other, very generally fails to exert an appreciable control over the progress of the disease. It is well known that bleeding, general and local; cathartics; and sedatives, have been used without any apparent benefit.

When the disease has occurred in districts naturally malarious, quinine and stimulants have been freely used; and if I remember correctly, both were used in most of the cases reported by Dr. UPHAM, but with no apparent influence over the progress of the disease. Calomel has also been used, both in cathartic and alterative doses; but with no more success. Indeed the rapid progress of the disease affords not sufficient time to gain any important alterative influence from the mercurial preparations. And if the special character of the inflammation in these cases, is asthenic or allied in nature to pyæmia, as I have already suggested, mercurialization as well as depletion, is directly contra-indicated. The clear indications for treatment in such a grade of inflammation, are to increase the contraction of the capillaries of the inflamed part for the purpose of retarding the accumulation of blood in them; and to change the aplastic or septic condition of the blood, thereby preventing if possible, the rapid development of the suppurative process with effusion. Being satisfied, from much clinical observation, that the views of BROWN SEQUARD, in relation to the action of belladonna on the cerebro-spinal centre, are correct; we should regard that agent as one of the most efficient for accomplishing the first indication named; while to meet the second indication we must rely on those remedies found most efficient in erysipelatous and pyæmic inflammations, such as the tincture of the chloride of iron and the sulphites of soda and lime.

During the last six months, five cases of cerebro-spinal meningitis have come under my care. The first was a boy, about 12 years of age. He had complained of headache and weakness for one or two days; but the severe characteristic symptoms did not commence until Saturday evening, and he died the following day in the afternoon. I saw him first, late on Saturday evening. I directed ice to his occipital and cervical regions; opened the bowels freely by a mercurial purge, and followed it with iodide of potassa. The next morning, he was visited by my colleagues, Professors ANDREWS and JOHNSON, who advised the use of quinine. The loss of consciousness and difficulty of deglutition, however, prevented the exhibition of more than a single dose.

The second case was an adult, female, and the mother of several children. After having felt some lassitude and indisposition for one or two days, she had a moderate chill, followed by some febrile reaction; severe pain in the back of the head, neck, and shoulders; with some convulsive movements and rigidity of the extremities. Alarmed at these symptoms, I was sent for in great haste, but did not reach the patient until about three hours had elapsed. I found her with moderate heat of the skin; an anxious expression of countenance; a small and frequent pulse; rigidly contracted condition of the muscles on the posterior part of the neck, causing the head to be drawn a little back, the jaws stiff, and deglutition difficult. The muscles of the arms were in a similar state of rigid contraction. All attempts to move the head and shoulders greatly aggravated the sufferings of the patient. The death of the boy, only a few days previously, had caused me to reflect much on the nature of the supposed inflammation in these cases, and I determined to put this patient directly on the use of the *sulphites* with belladonna. I accordingly directed 15 drops of the tincture of belladonna and half a drachm of sulphite of lime to be given at once, and repeated in half an hour, after which they were to be taken every hour, until the muscular rigidity ceased, or the specific effects of the belladonna were visible on the pupils of the eyes; after which the interval was to be extended to four hours. Ice was applied to the neck and occiput.

Under this treatment the muscular rigidity, pain, and fever soon began to abate, and in 24 hours all the severe symptoms were relieved, except stiffness of the neck and giddiness, with some pain whenever attempts were made to move the head. Under the moderate use of the same remedies, she continued to improve; and in four or five days was able to sit up. Her limbs, however, remained weak, and she was troubled with some unsteadiness in walking for ten or twelve days.

The third case was a boy, only two years old, in the same neighborhood with the second case, and was under treatment at the same time. The symptoms of cerebro-spinal inflammation were well marked, and the treatment the same as in the preceding case, only adapting the doses to the age of the patient. The relief was prompt and permanent, the child recovering fully in a week.

The fourth case was an adult, male, naturally athletic and healthy. He had been employed for some time as tender of a bridge, over the North Branch of the Chicago River: and that stream was at the time in a very foul and offensive condition. He was atacked in the night with a well-marked chill, followed by pain in the back of the head, neck, and shoulders; stiffness of the muscles of the neck, and frequent severe cramps in the muscles of the legs and forearms. When I was called to see him in the morning, his expression of countenance was haggard and anxious; his pulse small, frequent, and compressible; tongue moist; skin covered with moisture; mind depressed and taciturn; with considerable pain and stiffness in the neck, but, at that time, no cramps in the extremities. Learning that the patient had had a well-marked chill, followed by fever and pain, while he was then perspiring, I too hastily inferred that the case was one of irregular and severe malarious fever; an inference which would have been corrected readily had I given due attention to the locality of the pains and the condition of the muscles of the neck and extremities. As it was, however, I ordered the patient six powders, each containing sulphate of quinine, 3 grs., sulphate of morphia, $\frac{1}{3}$ gr., and calomel 2 grs., one to be given every three hours.

Before they were all taken, he became so furiously delirious that no treatment could be continued, and he died in little more than 48 hours after the commencement of the attack.

The fifth case occurred about two weeks since. The patient was a well-known citizen, aged about 50 years. On going out to his business in the morning, he felt some stiffness of the back of his neck, with an approach to vertigo or a slight sense of unsteadiness in walking. These symptoms were too slight to attract much attention during the day. About 5 o'clock P.M., he stepped into the office of a friend, where he suddenly became so affected with a sense of vertigo and exhaustion that he came near falling. He recovered his steadiness in a few minutes, and, accompanied by his friend, returned to his residence. During the evening he was chilly, and complained of severe pain in the back of his head, neck, and shoulders, with constant rigidity of the muscles of the neck.

These symptoms so rapidly increased in severity, that at 3 o'clock in the morning I was called out of bed to visit him. I found him with a countenance expressive of extreme anxiety and suffering; skin hot, but covered with perspiration; pulse small, frequent, and firm under pressure; tongue slightly coated; head drawn back and fixed firmly in its position by a rigid contraction of the muscles of the neck and jaws. He complained of intense pain in the back of the head and neck, extending in severe sharp paroxysms to the shoulders and right side of his chest. Every attempt to move the head or shoulders so aggravated these paroxysms of pain as to literally terrify the patient.

The skin being covered with perspiration, caused as the patient alleged by the severity of his pains, I did not deem it prudent to apply ice to the neck and occiput; but on the contrary ordered the application of cloths, kept constantly wet in a tepid infusion of aconite leaves. Internally, I directed 20 grs. of sulphite of soda, dissolved in a dessert spoonful of mint water, with 12 drops of the tincture of belladonna, every half-hour, until three doses were taken, and then once an hour, until my next visit. I also directed a powder containing 3 grs. of calomel and 6 grs. of Dover's powder, to be given every three hours. I visited the patient again in five hours. All my directions had been faithfully executed.

The second dose of calomel and Dover's powder caused slight vomiting, by which a part of it was rejected. On this account no more of them were given.

The only important changes in the condition of the patient were, a slower pulse; a more cheerful state of mind; and a subsidence of those severe paroxysms of pain through the shoulders and chest. The head was still firmly retracted, with pain in the neck, greatly aggravated by the slightest motion. I directed the sulphite of soda and belladonna to be continued every hour and a-half, and also, the narcotic fomentations to the neck. The patient continued to improve gradually through the day; and at night I directed the interval between the doses of the sulphite of soda and belladonna to be increased to two hours; and give three compound cathartic pills to move the bowels. On the following morning, I found the patient cheerful; the skin natural; pulse 85 per minute and soft; no pain while entirely still, or when slowly and cautiously rotating the head; but still inability to bring the head forward, or to indulge in any active movements without suffering. The pupils were moderately dilated by the belladonna, but there had been no movement of the bowels. I ordered a bottle of liquid citrate of magnesia to be taken in divided doses until it should operate, while the sulphite of soda and belladonna were continued every three hours. During the succeeding 24 hours, the bowels moved freely three times, and the patient continued steadily to improve; though he complained of much lassitude and general weakness.

I continued the sulphite of soda and belladonna every six hours for two days longer, with nourishment; at which time the patient was able to leave his bed, though still feeling difficulty in bending the head forward, and some unsteadiness in walking.

I then directed a prescription consisting of bromide of ammonium, disolved in syrup of wild eherry bark, to be taken three times a day; and the recoveey has since become complete. It must be remembered that during the time that these cases were occurring, erysipelas was unusually prevalent in the city; and the general sanitary condition ot the whole city bad. The treatment of those few cases is not sufficient to test the value any of the remedies used. But if the inflammation, in these epidemic and rapidly fatal cases of cerebro-spinal meningitis, is of that specific character, which renders it analagous to erysipelas or pyæmia, as we have already suggested, then certainly, we must look for remedies chiefly to such articles as will counteract the septic or suppurative tendency, with such narcotics as diminish the morbid sensibility, by contracting the cerebro-spinal capillaries. Of the first class of articles, we know of none more reliable, or capable of being more rapidly introduced into the system than the tincture of chloride of iron, and the sulphites of soda and lime, and the chlorates. Of the latter class, the preparations of belladonna and strammonium are doubtless most efficient. But for any class of remedies to be efficient in a disease so rapid in its progress as cerebro-spinal meningitis, they must be administered early and efficiently.

